# Sexual and gender identity and note-leaving among adult suicide decedents in the USA

**DOI:** 10.1192/bjo.2022.610

**Published:** 2022-12-21

**Authors:** Holly P. O'Rourke, Chanler D. Hilley, Emily Lowell, Connor M. Sheehan

**Affiliations:** T. Denny Sanford School of Social and Family Dynamics, Arizona State University, USA; Department of Psychological Science, Kennesaw State University, USA

**Keywords:** Gender and sexual minority (SGM), note-leaving, National Violent Death Reporting System (NVDRS), suicide, LGBTQ

## Abstract

**Background:**

Suicide is one of the leading causes of preventable death in the USA, representing a critical public health threat. Suicide risks differ for different populations. In particular, the sexual and gender minority (SGM) population remains at increased risk for suicide. One of the circumstances that may differ for SGM and non-SGM individuals is the propensity to leave a suicide note. Information regarding note-leaving may be helpful in informing suicide prevention and intervention.

**Aims:**

This study documents the differences in note-leaving in SGM individuals compared with non-SGM individuals, using recent data from the National Violent Death Reporting System (*N* = 98 515) and accounting for important covariates.

**Method:**

We fit a logistic regression model with SGM status and covariates predicting note-leaving in suicide.

**Results:**

SGM decedents were 1.508 times more likely to leave a note than their non-SGM counterparts, controlling for demographic, mental health and substance use covariates.

**Conclusions:**

These findings highlight the importance of tailoring suicide prevention and intervention efforts to meet the needs of SGM populations.

Suicide is one of the leading causes of preventable death in the USA, and although suicide is recognised as a critical public health problem, rates have been increasing in the past decade.^1,2^ Two factors that are important in suicide research are differences in risk rates and differences in circumstances. Researchers have historically noted that the risk of suicide is unequal throughout the population, as certain groups have substantially higher risk of dying by suicide.^3^ Sexual and gender minority (SGM) individuals in particular have significantly higher risk of dying by suicide than non-SGM individuals.^4–6^ Note-leaving is a circumstance surrounding suicide that has been used in suicide prevention efforts^7^ to address this public health problem. The circumstances surrounding suicide are defined by the National Violent Death Reporting System^8^ (NVDRS) as information regarding the decedent ‘reported or perceived in investigative reports’, typically immediately preceding the fatality. Suicide circumstances also differ across groups and include whether a suicide decedent left a suicide note.^9,10^ However, little is known regarding the circumstances surrounding suicide for SGM individuals. Given that SGM individuals have differential (increased) risk for suicide, they may also have important differences in circumstances surrounding suicide, such as note-leaving. In this paper, we utilise the NVDRS, a nationally comprehensive surveillance of suicide decedents in the USA, to examine if leaving a note before suicide varies between SGM and non-SGM individuals.

## Difference in risk and rates: SGM suicides

Evidence suggests that SGM individuals are at considerably higher risk of suicidal ideation, attempts and completed suicides than non-SGM individuals.^4,11–15^ Given the elevated risk, it is not surprising that past research has not only documented the higher risk of suicide among SGM individuals, but has also documented the factors that are commonly associated with increased risk of suicide and circumstantial differences in suicide among SGM individuals.^6,16^ This work has also stressed that adversities that are unique to SGM individuals, such as homophobic victimisation and bullying, are important factors that may elevate suicide risk. Thus, although researchers have credibly documented that SGM individuals are a high-risk group and have characterised the risk factors of suicide within the SGM population, extant research has not specifically analysed note-leaving circumstances as a primary outcome of interest for all SGM individuals while controlling for key covariates.

## Differences in circumstances: note-leaving

A sizable proportion of suicide decedents leave a suicide note, and this is a phenomenon that appears cross-culturally in several countries.^17–20^ Past research has analysed the content of suicide notes and found that they generally focus on major life stressors such as financial and relationship problems.^21–24^ Other research has used regional samples to analyse demographic characteristics that are linked to increased propensity of leaving a suicide note, but these studies have provided discrepant results, which may be a result of differing contexts of analysis and/or small samples.^17,25,26^ Based on a review of prior research, it has been argued that suicide decedents who do and do not leave suicide notes are relatively similar with respect to a host of demographic characteristics.^25^ However, since that review, researchers using the NVDRS have found that women, middle-aged individuals, those with intimate partner problems or financial problems, and those without substance use issues were more likely to leave a note.^9^ This systematic documentation of note-leaving at the national level was a fundamental step in better understanding who leaves suicide notes, but did not examine sexual orientation or gender identity. One study used the 2003–2014 years of the NVDRS to examine the relation between sexuality and note-leaving as a suicide circumstance separately for cisgender men and women, and found higher instances of gay and lesbian individuals leaving a suicide note.^27^ However, this descriptive finding excluded transgender and bisexual individuals from primary analysis, and relied on narrative coding to determine SGM status.

## The current study

The purpose of the present study is to address this gap in the literature by documenting whether SGM individuals are differentially likely to leave suicide notes compared with non-SGM individuals, while controlling for important covariates that past work has linked to suicide note-leaving.^9,^^10^ This study also updates and expands upon prior work with NVDRS data by investigating demographic differences in note-leaving across a longer (and more recent) period of time that includes more jurisdictions than previous studies, achieving a more complete understanding of recent note-leaving among suicide decedents in the USA. Based on prior research with cisgender individuals, we predicted that all SGM individuals would be more likely to leave a suicide note than non-SGM individuals, above and beyond the host of covariates that also predict suicide note-leaving.

## Method

### Data source and reporting procedure

Data for the present study were drawn from the NVDRS, a national surveillance that documents violent deaths in the USA. The NVDRS collects data on demographics, mental health, precipitating circumstances surrounding death and manner of violent deaths in the USA. The data-set provided to our team by NVDRS contained demographic, substance use, mental health and death circumstance information for adult (aged ≥18 years) suicide decedents. Data were provided from 37 US states from 2013–2017 (the years and states for which sexuality and gender identity information was available), resulting in a sample of *N* = 98 515.

### Measures

#### Suicide note-leaving

Note-leaving was the main outcome of interest. This variable was coded 0 (leaving no note or other recorded communication) or 1 (leaving a note or other recorded communication). Past research has used this classification of suicide note-leaving from the NVDRS.^9,^^10^

#### Gender and sexuality

Sexual orientation and transgender identity were measured with two separate variables. These variables were used to create a single binary variable classifying individuals as SGM or non-SGM. The original sexual orientation variable was coded as heterosexual, gay, lesbian, bisexual or unknown. The original transgender variable was binary (no/unknown/no available information versus transgender). The SGM variable created from these was the main predictor of interest and was ultimately coded as 0 (non-SGM) or 1 (gay/lesbian/bisexual and/or transgender), with unknown sexuality coded as missing. Thus, this variable was coded such that only individuals who were reported as positively identifying as gay, lesbian, bisexual or transgender were coded as SGM. A large proportion of individuals had unknown sexuality, resulting in relatively few cases with complete data on this variable (*n* = 18 594), which was addressed analytically by multiple imputation.

### Covariates

To keep the statistical model parsimonious and ensure accurate type 1 error levels by avoiding multicollinearity, covariates were chosen based on two criteria. Initial covariates were selected to be a subset of covariates from prior studies of note-leaving with the NVDRS data-set.^9^ First, covariates were only included in the final analysis if they had a significant tetrachoric correlation with the outcome of note-leaving. Second, certain covariates were excluded from the final analysis if they had high multicollinearity with other selected covariates (for example, multiple alcohol measures capturing the same construct).

#### Demographic characteristics

Analyses included five demographic constructs: sex, race, education, military status and age. Sex was coded as 0 (male) or 1 (female). Race was coded with White as the reference group, compared with Black/African American; non-Hispanic; other non-Hispanic, including multi-racial; and Hispanic. Education was coded with less than high school degree as the reference group, compared with high school diploma/GED, some college education and Bachelor's degree or higher. Military status was a binary variable coded as 0 (no military service) or 1 (military service). Age in years was included as a continuous covariate with no transformations.

#### Mental health variables

Mental health circumstances were measured by suicidal thought history (‘Victim had a history of suicidal thoughts or plans’), suicide attempt history (‘Victim had a history of attempting suicide before the fatal incident’) and mental health problems (‘Victim had been identified as currently having a mental health problem’). Substance use was measured by alcohol dependence or alcohol problems (‘Person has alcohol dependence or alcohol problem’). Each of these variables was binary (no/unknown/no available information versus yes).

### Statistical analyses

To examine the role of SGM identity on suicide note-leaving, we employed a multiple logistic regression model for the binary outcome. We examined the influence of SGM identity (the focal predictor) on note-leaving (the outcome), accounting for the covariates described above. All analyses were conducted in R software, version 4.0.2.^28^ As mentioned above, the creation of the SGM variable resulted in 18.51% complete data for that variable (all other variables had ≥80% complete data). To handle the missing data, multiple imputation was implemented with the MICE package in R software version 4.0.2.^28^ Five data-sets were multiply imputed with a maximum of 25 iterations for the burn-in period, and a random number seed of 500. All analyses following multiple imputation were then conducted on the pooled data with the MICE package in R.

## Results

Descriptive statistics for demographics and variables of interest are given in Table 1.

**Table 1:** Descriptive statistics, National Violent Death Reporting System data (2013–2017)

	*n* (%)^a^	
	Original	Multiple imputation
Variables of interest		
SGM (reference: non-SGM)	969 (5.31)	37 359 (7.58)
Suicide note (reference: no, not available, unknown)	30 477 (30.94)	152 385 (30.94)
Demographic characteristics		
Female (reference: male)	22 392 (22.73)	111 975 (22.73)
Military veteran (reference: not a military veteran)	16 761 (17.86)	87 840 (17.83)
Race/ethnicity		
Non-Hispanic White (reference)	82 512 (83.87)	413 054 (83.86)
Non-Hispanic Black	5696 (5.79)	28 530 (5.79)
Hispanic	5602 (5.69)	28 070 (5.70)
Non-Hispanic other	4576 (4.65)	22 921 (4.65)
Educational attainment		
Less than high school (reference)	11 971 (13.60)	66 609 (13.52)
High school diploma/GED	37 007 (42.04)	205 290 (41.68)
Some college education	22 514 (25.58)	126 615 (25.70)
Bachelor's degree or higher	16 538 (18.79)	94 061 (19.10)
Age, mean (s.d.)	47.72 (17.78)	47.71 (17.78)
Mental and substance use issues		
Suicidal thought history (reference: no, not available, unknown)	29 381 (29.82)	146 905 (29.82)
Suicide attempt history (reference: no, not available, unknown)	18 145 (18.41)	90 725 (18.42)
Mental health problems (reference: no, not available, unknown)	44 284 (44.95)	221 420 (44.95)
Alcohol problem (reference: no, not available, unknown)	16 667 (16.92)	83 335 (16.92)

SGM, sexual and gender minority.

a. Percentage out of complete data for each variable.

Descriptive statistics are included for both the original data-set and the five imputed data-sets. The proportion of SGM individuals increased slightly in the pooled imputed data; otherwise, the proportions of each category were consistent across the original and imputed data. The majority of suicide decedents were male, White and not members of the military. Decedents most commonly had a high school diploma or higher. Almost half of all decedents (44.95%) had a history of mental health problems, an expected proportion given the outcome being studied.

Results from the pooled logistic regression are presented in Table 2.

**Table 2:** Coefficients from logistic regression predicting suicide note-leaving from the National Violent Death Reporting System (2013–2017)

Predictor	*b-*value	s.e.	*p-*value	Odds ratio	95% CI for OR
SGM (reference: non-SGM)	0.411	0.075	0.003	1.508	(1.245–1.827)
Demographic characteristics					
Gender (reference: male)	0.283	0.017	<0.001	1.327	(1.284–1.373)
Age	0.003	0.004	<0.001	1.003	(1.002–1.004)
Race/ethnicity (reference: non-Hispanic White)					
Non-Hispanic Black	−0.542	0.036	<0.001	0.582	(0.543–0.624)
Hispanic	−0.267	0.033	<0.001	0.766	(0.718–0.817)
Non-Hispanic other	−0.182	0.034	<0.001	0.834	(0.779–0.892)
Educational Attainment (reference: less than high school)					
High school	0.386	0.025	<0.001	1.470	(1.399–1.546)
Some college education	0.535	0.027	<0.001	1.708	(1.621–1.801)
Bachelor's degree or higher	0.808	0.028	<0.001	2.243	(2.122–2.371)
Military veteran (reference: not a military veteran)	0.026	0.020	0.178	1.027	(0.988–1.067)
Mental and substance use issues					
Suicidal thought history (reference: no past suicidal thoughts)	0.135	0.016	<0.001	1.114	(1.109–1.181)
Suicide attempt history (reference: no past suicide attempts)	0.068	0.019	<0.001	1.070	(1.031–1.112)
Mental health problems (reference: no mental health problems)	0.017	0.015	0.256	1.017	(0.988–1.048)
Alcohol problem (reference: no alcohol problems)	−0.214	0.020	<0.001	0.807	(0.776–0.839)
Intercept	−1.483	0.033	<0.001	0.227	(0.213–0.242)

The *b*-value indicates the logistic regression coefficient. The odds ratio indicates the exponentiated *b*-value. SGM, sexual and gender minority.

Each estimate is reported in the context of controlling for all other variables. Unstandardised slope coefficients are reported in addition to odds ratios. Our primary predictor of interest, SGM identity, was a significant predictor of suicide note-leaving (*b* = 0.411, *P* = 0.003) such that the odds of leaving a note before suicide for SGM decedents were 1.508 times higher than that of non-SGM decedents (95% CI 1.245–1.827).

Each of the social demographic characteristics except military veteran status was also a significant predictor of note-leaving. The odds of leaving a note before suicide were 1.327 times higher for females than for males (95% CI 1.284–1.373). The odds of leaving a note before suicide increased by a factor of 0.003 for each additional year in age (95% CI 1.002–1.004). Decedents who were non-Hispanic Black (odds ratio 0.582, 95% CI 0.543–0.624), Hispanic (odds ratio 0.766, 95% CI 0.718–0.817) or other non-Hispanic ethnicity including multiracial ethnicity (odds ratio 0.834, 95% CI 0.779–0.892) were less likely to have left a suicide note than White decedents. Decedents with a high school diploma or GED (odds ratio 1.470, 95% CI 1.399–1.546), some college education (odds ratio 1.708, 95% CI 1.621–1.801) or Bachelor's degree or higher (odds ratio 2.243, 95% CI 2.122–2.371) were more likely to have left a suicide note than decedents who had less than a high school diploma or GED, with odds of leaving a suicide note increasing for each level of additional educational attainment.

Most of the mental health and substance use issues covariates (except for mental health problems) included in the model were also significant predictors of note-leaving. The odds of leaving a note before suicide for decedents with a history of suicidal thoughts were 1.144 times that of those with no history of suicidal thoughts (95% CI 1.109–1.181). The odds of leaving a note before suicide for decedents with a history of any suicide attempt were 1.070 times higher than that of decedents with no history of suicide attempt (95% CI 1.031–1.112). Decedents with mental health problems were not significantly more or less likely than decedents without mental health problems to leave a suicide note, after controlling for covariates (odds ratio 1.017, 95% CI 0.988–1.048). Finally, decedents with alcohol problems were less likely to leave a note before suicide than decedents without alcohol problems (odds ratio 0.807, 95% CI 0.776–0.839); specifically, there was a 19.3% decrease in odds of leaving a note before suicide for decedents with alcohol problems compared with decedents without alcohol problems.

## Discussion

Suicide remains an important cause of preventable death in the USA, and SGM individuals experience heightened risk for suicide attempts in their lifetimes.^4–6^ Thus, efforts to understand circumstances surrounding suicide, including whether a decedent left a suicide note, are of critical public health concern. In this study, we built upon prior literature by investigating differences in suicide note-leaving among SGM and non-SGM individuals, while also controlling for a host of covariates that have been identified as key predictors of suicide note-leaving in prior research.^9,^^10^ The present study supports prior research that SGM individuals are more likely to leave a note, including in the primary analyses data (although limited) on bisexual individuals and transgender individuals that have not been incorporated in prior studies with NVDRS data. Additionally, the present study made use of more recent data and included more jurisdictions than prior studies, providing an up-to-date picture of suicide circumstances for SGM individuals. We found that SGM suicide decedents were more likely to leave suicide notes than non-SGM individuals even when controlling for demographic, mental health and substance use variables. The inclusion of these covariates also effectively enabled the replication of prior work on note-leaving more generally, using earlier waves of NVDRS.^9,^^10^ Additionally, findings relating to the covariates from the present study were largely consistent with prior studies that used NVDRS data.

### Implications for preventive interventions

Understanding whether SGM individuals leave suicide notes at higher rates than non-SGM individuals may provide important insight that can be used for early detection and targeted prevention efforts to those most vulnerable to suicide. Indeed, a scoping review of the published studies that used NVDRS data highlighted that the potential for the NVDRS to inform prevention and intervention efforts remains largely untapped.^29^ A recent meta-analysis found that across 50 years of research, suicide interventions generally had small, inconsistent effects, and researchers concluded that future interventions could be more powerful by targeting suicide correlates and risk factors.^30^ In a summary of systematic reviews, Van der Feltz-Cornelis and colleagues^31^ highlighted that understanding the differential suicide risk of vulnerable populations, which include SGM individuals, is key to the effective prevention of suicides and must be incorporated at multiple levels. Such methods may include, for example, training on the differential risk of SGM for both practitioners and community-level stakeholders; targeted messaging for SGM individuals specifically in suicide prevention programmes and mental health treatments; and generally improving access to mental healthcare for SGM individuals, although this warrants a larger discussion related to global mental health in SGM individuals. However, little is currently known about the precipitating circumstances surrounding suicides in the SGM population, and a more thorough, nuanced understanding of the circumstances that contribute to suicide in SGM individuals will help to inform a coordinated suicide prevention strategy.

Additionally, effective preventive interventions should be driven by both theory and empirical evidence. The interpersonal theory of suicidal behaviour^32^ suggests the importance of two key factors in predicting suicidal behaviour: thwarted belongingness (i.e. an ‘unmet need’ to feel like one belongs) and perceptions of burdensomeness (i.e. feeling that one's existence is a burden to others). SGM individuals, in particular, may face experiences of rejection and discrimination as a result of their gender identity and/or sexual orientation (i.e. aspects of minority stress), and these external experiences may then lead to internalised SGM stigma.^33,34^ For example, a study using psychological autopsy in Australia found that lack of acceptance was higher among SGM suicide decedents than their living case–control peers.^35^ Thus, the content of suicide notes from SGM individuals may highlight specific ways in which SGM individuals may experience burdensomeness or lack of belongingness, and may have implications that make the interpersonal theory of suicidal behaviour (and other theories) more culturally relevant to SGM populations and, ultimately, lead to interventions with stronger theoretical groundings. Themes reflected in the contents of these notes may provide theoretical contributions to prevention strategies that are more relevant to the lived experiences of SGM and, thus, enable the development of interventions that more effectively reduce risk of suicide among this population.

Examining precipitating circumstances surrounding suicide, some of which may be captured in the content of suicide notes and relevant to psychological autopsies,^36^ may provide essential information for targeted prevention and intervention efforts to decrease risk of suicide for SGM populations. Additionally, the period of time immediately preceding suicide, in which suicide notes may be written, may be a critical period for effective intervention. Indeed, researchers have recently attempted to develop tools for suicide prevention by using risk assessment questions that involve suicide notes and the themes that appear in them (i.e. feelings of hopelessness; sadness; burdensomeness; lack of connection; intent to die and intensity, duration and frequency of suicidal thoughts).^7^ The assessment of note-leaving behaviours is important for intervention because prior research indicates key demographic differences in those who leave a note versus those who do not. Researchers can use those demographics to assess certain individuals for note-leaving before suicide with more specificity, thereby potentially preventing suicide.

### Limitations and future directions

We acknowledge several limitations of this study, some of which are related to the reporting system.^8^ Despite now including data from all 50 US states, data from the NVDRS are not nationally representative, and the states that chose to participate varied in their participation over time. Additionally, given the extreme variability in the reporting process and timing of reporting, the precipitating circumstances surrounding some suicides are unknown. Related to this, a general measurement issue of the NVDRS is the categorisation of ‘unknown’ or ‘unavailable’ status with known ‘no’ responses, precluding researchers from excluding low-quality cases from confirmed negative cases. Furthermore, the NVDRS data-set is limited in its generalisability with respect to suicide behaviour, in that it does not capture note-leaving for individuals who have attempted suicide.

Additionally, several variables related to sexuality and gender (which are more recently added variables) have high missing data rates, which we have addressed in the present study by multiply imputing data-sets and reporting the pooled results. As noted in the measures section, several variables (including SGM identity and mental health variables) also relied on the reports of individuals who knew the decedent, rather than their healthcare professionals or medical records. In commentaries on prior studies, specific attention has been paid to the coding of the data used to create the SGM variable, noting that reporting of LGBTQ identity may or may not be inflated in the NVDRS (i.e. because of the proportion of ‘unknown’ coding in lesbian/gay/bisexual and transgender variables),^37,38^ and thus our results should be interpreted in view of these limitations. Potential quality issues could be that reporters who knew the individual are unwilling or unable to disclose identity features related to sexuality and gender for a variety of reasons.

Along this same line of reasoning, we considered incorporating sex as a moderator of our main effect of interest, but ultimately chose not to, given the potential conflation in reporting. Sex was recorded as ‘the victim's sex at the time of the incident’, and given the lack of circumstantial data, we were unable to ascertain how transgender individuals would have been coded (e.g. sex recorded as male may have indicated any of the following: cisgender males, individuals assigned female at birth who identified as males and individuals assigned male at birth who identified as females, as well as other transgender or intersex identities). Thus, we considered the SGM × sex interaction to be uniquely inappropriate to examine, given the lack of coding clarity in the current data. This may be an important consideration for future research, as well as for collecting future waves of data in NVDRS.

Further work is still needed to elucidate contextual differences in the precipitating circumstances surrounding SGM suicides. To best inform prevention efforts, future research may consider additional analyses of the content of suicide notes left by SGM decedents, and whether it varies compared with the content of suicide notes left by non-SGM decedents. In previous content analyses of law enforcement and medical examiner reports of youth suicides, Ream^39^ found that family and peer rejection, bullying and romantic breakups were more prevalent among certain SGM subgroups than non-SGM decedents. The content of suicide notes have already been influential in informing theoretical perspectives on suicidal behaviour (e.g. the interpersonal theory of suicidal behaviour),^32^ and may also provide more proximal information regarding the precipitating circumstances surrounding some SGM suicides so as to better inform prevention efforts tailored to diverse SGM populations.

In conclusion, suicide is a serious public health concern in the USA, warranting significant investment by researchers toward major prevention and intervention efforts. SGM individuals are at higher risk for suicidal ideation and suicide attempts, and extant research has not yet established whether disparities exist between SGM and non-SGM individuals regarding circumstances surrounding suicide behaviour. Contributing to this research, the present study examined SGM identity as a predictor of suicide note-leaving, using data from the USA's NVDRS. We found that SGM individuals had higher odds of suicide note-leaving than their non-SGM peers, controlling for demographic, mental health and substance use variables. This study also extends prior work examining these covariates by replicating prior findings over a longer course of time, and with data from jurisdictions that have commenced participation in the NVDRS after the publication of prior findings. Although research is still needed to understand the complexities of SGM suicides, our and previous findings suggest that suicide prevention and intervention strategies need to be tailored or targeted to more effectively address SGM suicides, in which leaving a note before suicide can play a key role in strategy development.

## Data Availability

Data analysed in this study were collected via the National Violent Death Reporting System, which is administered by the Centers for Disease Control and Prevention (CDC). Researchers may request access to the de-identified Restricted Access Database from the CDC (https://www.cdc.gov/violenceprevention/datasources/nvdrs/index.html).
